# The first Neolithic boats in the Mediterranean: The settlement of La Marmotta (Anguillara Sabazia, Lazio, Italy)

**DOI:** 10.1371/journal.pone.0299765

**Published:** 2024-03-20

**Authors:** Juan F. Gibaja, Mario Mineo, Francisco Javier Santos, Berta Morell, Laura Caruso-Fermé, Gerard Remolins, Alba Masclans, Niccolò Mazzucco

**Affiliations:** 1 Milà i Fontanals Institution, Spanish National Research Council, Barcelona, Spain; 2 Museo delle Civiltà di Roma, Piazza Guglielmo Marconi, Rome, Italy; 3 Centro Nacional de Aceleradores (Universidad de Sevilla, CSIC, Junta de Andalucía), Sevilla, Spain; 4 Instituto Patagónico de Ciencias Sociales y Humanas (IPCSH-CONICET), Puerto Madryn, Prov. de Chubut, Argentina; 5 Regirarocs, Organyà, Lleida, Spain; 6 Dipartimento di Civiltà e Forme del Sapere, Università di Pisa, Pisa, Italy; New York State Museum, UNITED STATES

## Abstract

Navigation in the Mediterranean in the Neolithic is studied here through the boats that were used, the degree of technical specialisation in their construction and, above all, their chronology. After a brief explanation of the exceptional site of La Marmotta, the characteristics and chronology of the five canoes found at the settlement and one of the nautical objects linked to Canoe 1 are discussed. This will allow a reflection on the capability of Neolithic societies for navigation owing to their high technological level. This technology was an essential part in the success of their expansion, bearing in mind that in a few millennia they occupied the whole Mediterranean from Cyprus to the Atlantic seaboard of the Iberian Peninsula.

## 1. Introduction. Navigation in the Mediterranean in the Neolithic

Many of the most important civilisations in Europe originated on the shores of the Mediterranean Sea. Phoenicians, Greeks, Romans and Carthaginians plied that practically enclosed sea to move rapidly along its coasts and between its islands. In different historical times, the Mediterranean was a space in which to travel and a means of communication. However, one of the main migratory phenomena in history took place in the Neolithic, when farming communities began to spread around Europe and North Africa. Although the beginnings of the Neolithic are documented in the Near East in about 10,000 cal. BC, communities from that region gradually occupied the whole Mediterranean around 7500–7000 cal. BC and reached the coasts of Portugal in about 5400 cal. BC [1].

It is clear that the Mediterranean Sea must have often been used for travel, as boats allowed rapid movements of population, contacts and exchange of goods. This is seen not only in the vessel or other watercrafts, the subject of this paper, but also in the location of the first Neolithic settlements on islands or near the sea. For this reason, several researchers [2, 3] have proposed that the first farming communities must have travelled by sea, by means of short voyages following the coastline.

Obviously, those groups did not set sail without knowing what lay beyond the horizon they saw from their shores. Their knowledge about the maritime routes began to be acquired by Mesolithic groups, and possibly before, and was transmitted and perfected from generation to generation.

Much of the first indirect and direct evidence of maritime travel in Europe has been found at Mesolithic sites. Sea voyages explain the occupation during this period of Cyprus, Corsica, Sicily and Greek islands like Icaria, Lemnos and Melos [4–15]. Canoes discovered at several sites open a window to past navigation. They have been preserved under water (in lakes and lagoons) or in very humid sites (peat bogs). Their documentation reveals the types of boats that were used and their building techniques. Some of the most notable examples of Mesolithic canoes have been found at Noyen-sur-Seine and Le-Codray-Montceaux-Nandy, in France; Dümmerlohausen and Stralsund-Mischwasserspeicher, in Germany; Pesse, in Holland; Tybrind Vig, Lystrup and Praestelyng II-Baden in Denmark, and Hotiza, in Slovenia [16–23].

Although at that time the canoes were usually made from pine trunks (*Pinus sylvestris*), other species were used, such as poplar (*Populus tremula*) in the case of the canoes at Lystrup, oak (*Quercus* sp.) and alder (*Alnus* sp.) at Dümmerlohausen and Hotiza, and lime (*Tilia* sp.) at Tybrind Vig and Stralsund-Mischwasserspeicher (S1 File).

They were monoxylous canoes or dugouts made from a single trunk, of very different sizes. Some were small, practically for a single person, as at Pesse (7920–6470 BC), 3m long, or at Noyen-sur-Seine (7190–6540 BC), 4.5m long. Others were larger, like Canoes I and II at Lystrup (5200–5000 BC), 6-7m in length, one at Le-Codray-Montceaux (7240–6720 BC), and Canoe 2 at Stralsund-Mischwasserspeicher (4800–4700 BC), 8m long, and those at Tybrind Vig (4300–4100 BC), 10m long. Together with these, other nautical elements have sometimes been found, such as oars. These have been documented at the Danish sites of Tybrind Vig, Holmegaard and Ulkestrup Lyng [16, 17, 24]. Finally, remains of combustion identified inside some of the canoes shows that they were made by burning the middle of the trunks, which speeded up the work of hollowing them as it was thus easier to cut out the wood.

This model of dugout canoe, of varying sizes and made of different wood, using combustion of the trunk to hollow it, continued in the Neolithic. The canoes of La Marmotta are currently the only boats known at Neolithic sites in the Mediterranean basin. However, numerous canoes dated in more recent periods have been found in other countries [18, 20, 25]. Thus, for instance, the canoes at Seeland, Denmark (3640 and 2920 BC), 7m long, were made of alder (*Alnus* sp.). The one at Bevaix, Switzerland (3500–3030 BC), 8.27m long, was made of pine (*Pinus* sp.). Canoe 1 at Stralsund-Mischwasserspeicher, Germany (3858 BC) was 12m long and made from a lime trunk (*Tilia* sp.). Finally, canoes from the French sites of Paris-Bercy (2890–2510 BC), 6 and 8m long, and one from Charente, 5.56m long, (3650–2900 BC), were made from oak (*Quercus* sp.) [18, 22, 24, 26, 27].

### 2. La Marmotta: The earliest Neolithic lakeshore village in the central Mediterranean

Taphonomic processes and preservation conditions in general mean that we normally obtain a very biased and limited picture of the archaeological remains left by prehistoric communities. This image changes drastically at sites conserving many of the biotic remains that usually disappear because of bacterial activity. The observation of objects made of wood, textiles, basketry, cordage and animal skins completely alters our idea of those communities. The lakeshore site of La Marmotta is one of those cases in which the exceptional conservation of the archaeological artefacts validates a reflection on the numerous materials they used, their skill in working them and the high technical level reached by Neolithic societies.

**Fig 1:**
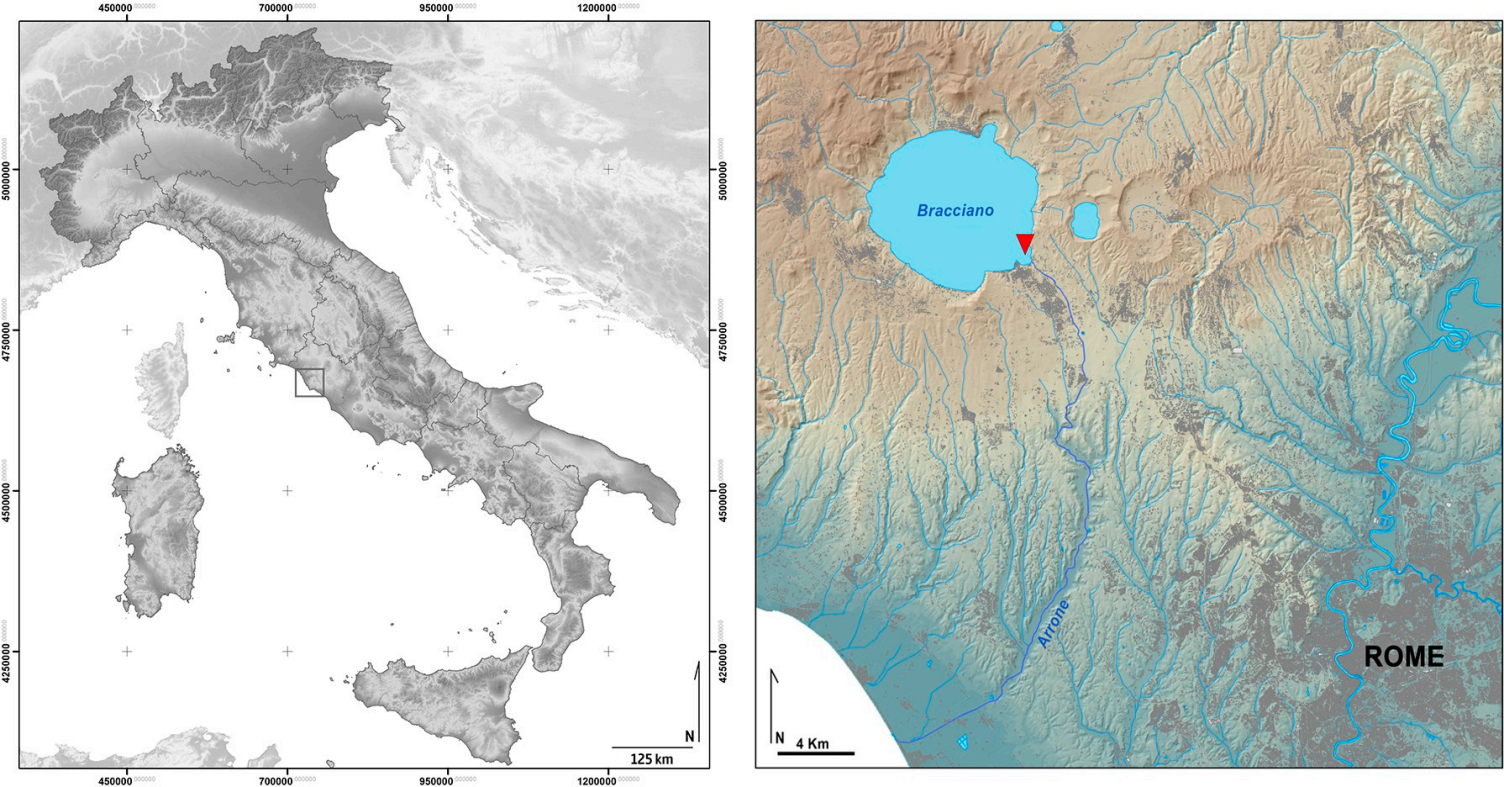
Location of La Marmotta in the Italian Peninsula.

The first evidence of the site, under Lake Bracciano (Anguillara Sabazia, Lazio, Italy) was found in 1989. It was then excavated from 1992 to 2006 under the supervision of the Superintendency of the Museo delle Civiltà “Luigi Pigorini”, headed by Dr Fugazzola (Fig 1). Another small excavation was carried out in 2009, the last fieldwork at the site.

**Fig 2:**
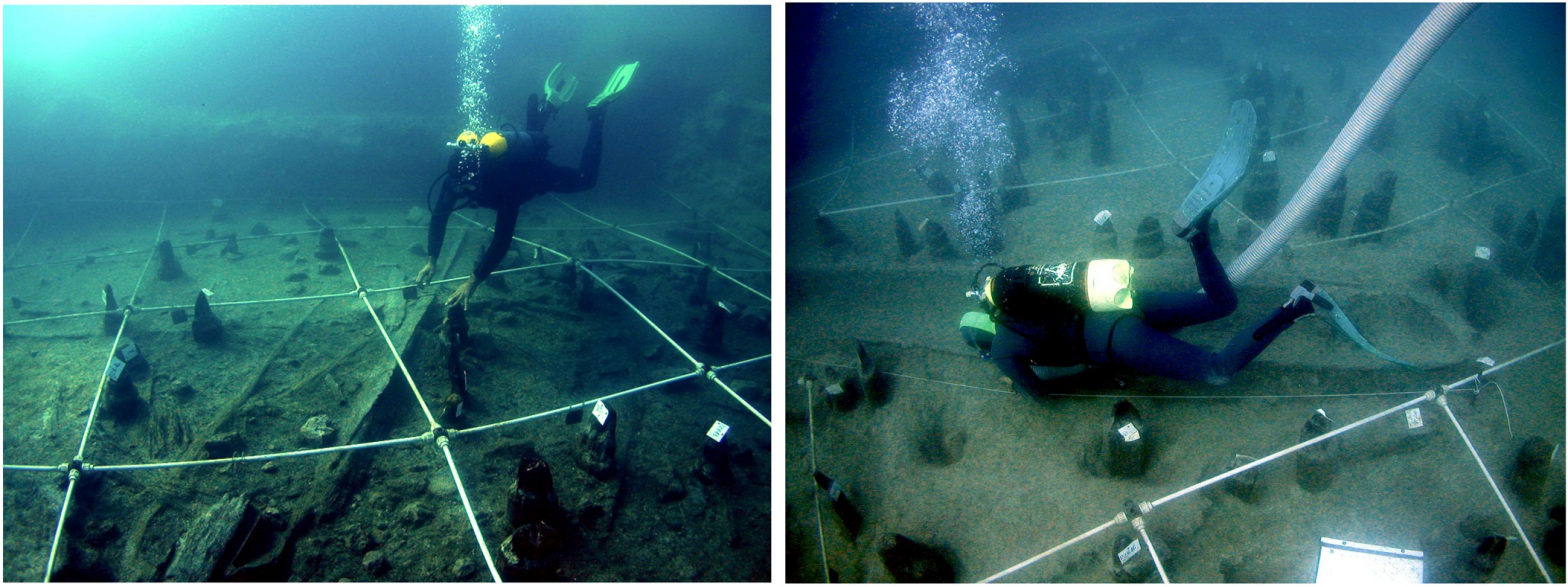
Excavation of Canoe 5.

The archaeological area is now about 300m from the modern lakeshore, at a depth of 11m (8m of water and 3m of sediment). This is a natural form of protection, safely under water and earth (Fig 2). Lake Bracciano is connected to the Mediterranean Sea by the River Arrone, over a distance of 38km.

Three main levels have been defined. While Level II, corresponding to the foundation of the settlement, is linked to the presence of impressed ware and, sporadically, incised ware (reminiscent of the Basi-Pienza style), Level I represent the most recent phase at the settlement and is associated with painted and incised pottery in the Sasso-Fiorano style. Finally, the level known as “*Chiocciolaio*” is the uppermost layer in the sequence and reflects the abandonment of the settlement [28, 29].

**Fig 3:**
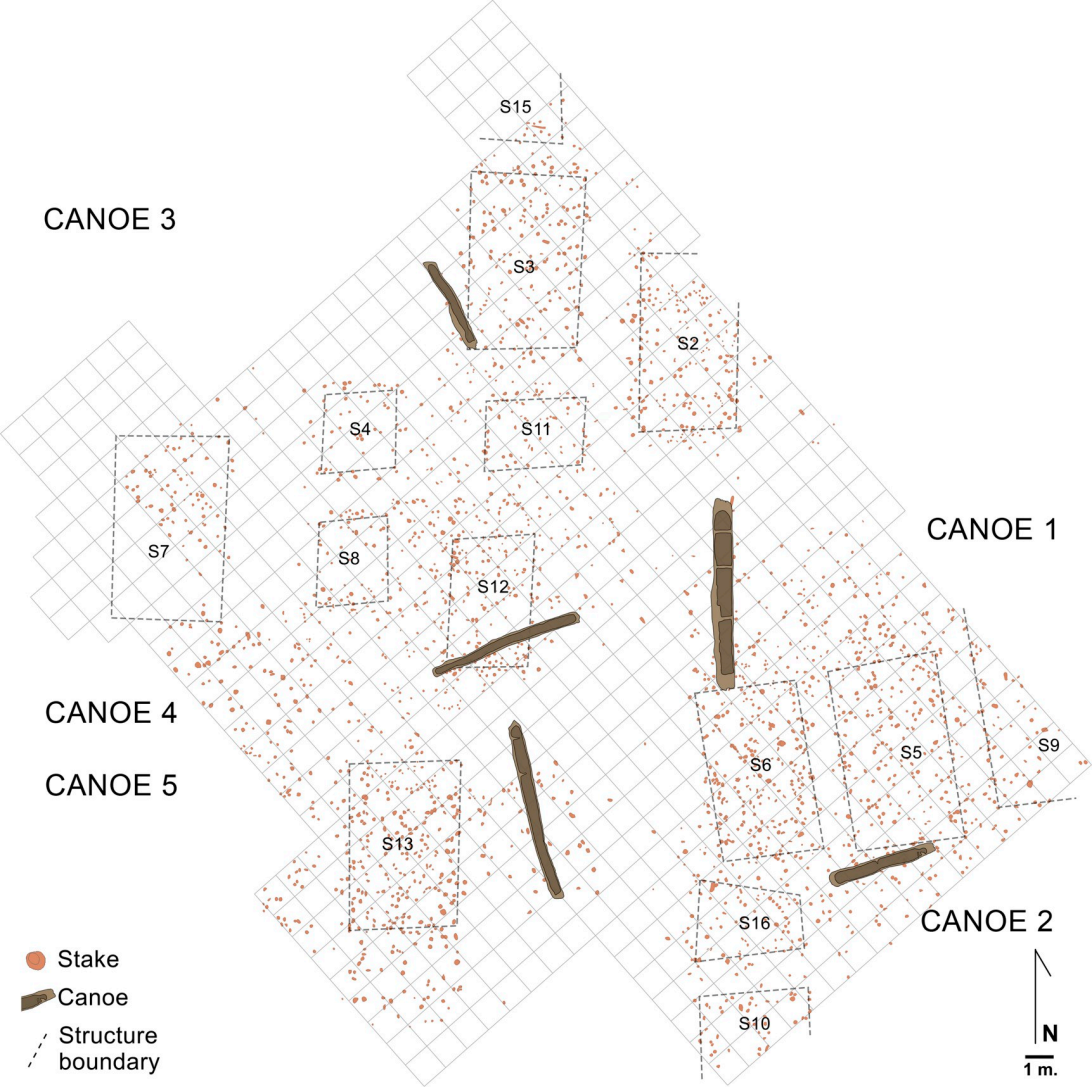
Plan of the settlement of La Marmotta with the five canoes next to some of the houses (Picture: Gerard Remolins).

During the excavation, 3,400 piles supporting the structure of the dwellings were found, as well as remains of the walls, made with wattle and daub, the roofs, consisting of the stems of different kinds of plants, and some wooden floors, made with timber or bark. Their positions were able to define a group of 14 possible rectangular dwellings with internal walls and a central hearth (Numbers 3, 4+8, 5, 6, 7, 11, 13 and 16 were excavated completely and 2, 9, 10, 14, 15 and 17 partially [30]). The houses were about 8 to 10m long and 6m wide. The five canoes found at La Marmotta were associated with some of the houses. Thus, Canoe 1 was next to Structure 6, Canoe 2 was near Structure 5, Canoe 3 near Structure 12, Canoe 4 next to Structure 3 and Canoe 5 next to Structure 13 (Fig 3).

Plant and animal remains are indicative of a community with a fully consolidated domestic economy. Domestic livestock make up about 75% of the minimum number of individuals documented at La Marmotta. These consist mostly of sheep and goats, together with fewer cattle and pigs. The faunal assemblage also includes two canine species of different size and a wide array of wild animals, including mammals (*Cervus elaphus*, *Capreolus capreolus*, *Bos primigenius*, *Vulpes vulpes*, etc.), birds, reptiles and fish [31].

In turn, 65% of the botanic remains correspond to different types of domestic cereals (*Triticum dicoccum L*., *Triticum monococcum S*., *Hordeum distichum*, *Hordeum vulgare L*. and *Triticum aestivum compactum e durum*). The other remains are legumes (*Pisum sativum*, *Lens culinaris*, *Lathyrus cicera/sativus*, *Vicia* cfr. *sativa*), several kinds of fruit (*Prunus spinosa*, *Ficus carica*, *Sambucus sp*., *Fragaria vesca*, *Rubus fruticosus*, *Corylus avellana*, *Quercus* sp., etc.) and plants used to make textiles, oil and dyes or with phytotherapeutic properties (*Linum usatissimum*, *Papaver somniferum*, *Carthamus lanatus* and *Silybum marianum*). The inhabitants of La Marmotta also collected fungi as tinder to light fires (*Fomes fomentarius*) or because of their medicinal effects (*Daedalopsis tricolor*) [32–34].

However, La Marmotta is also special because of the conservation of numerous and varied utensils and implements made from wood, basketry and textiles. Some examples are the bows, adzes, sickles, spoons, spindles, artefacts possibly related to working with textiles, wooden recipients and baskets, and, above all, the canoes [35, 36].

Together with the wooden implements, a large chipped lithic toolkit comprises 12,000 objects generally made with high-quality varieties of flint and to a lesser extent with obsidian. Heavy-duty tools are equally numerous: polished stone axes and adzes, querns, handstones, hammerstones and polishers. Some of those polishing stones display the marks left by the abrasion of axes and adzes, by sharpening bone and wooden tools, or making ornaments from shells, stones, wood, seeds, ceramics or the teeth/fangs of different animals.

The stones used to make these chipped and polished tools come from different provenances. Some flint is probably from the mines at Defensola in the Foggia region of Italy, obsidian from the islands of Palmarola and Lipari, and some hard stones used to make the axes and adzes come from the Alps [37, 38].

The ^14^C dates obtained from remains of charcoal and seeds, and the dendrochronological analyses of the piles supporting the houses, indicate the site was in use between approximately 5700 and 5150 cal. BC; that is to say, during an uninterrupted period of about 550 years. On the other hand, based on dendrochronological data, they seem to point to and uninterrupted use of the settlement for at least 250 years [30, 39] A new series of dates is focused on the analysis of short-lived samples (cereal grains from different levels) and some wooden artefacts, including the five canoes presented here.

## 3. The Marmotta canoes: True nautical engineering

Although it is difficult to estimate the area occupied by the settlement of La Marmotta, based on the results of the archaeological excavation, it is possible that a large part of the site remains unexcavated. We believe that there may be a larger number of boats still preserved under the waters of Lake Bracciano, and it is possible that they could be excavated in the future. To date, in the archaeological fieldwork that has been conducted, five canoes have been found [40–43].

**Fig 4:**
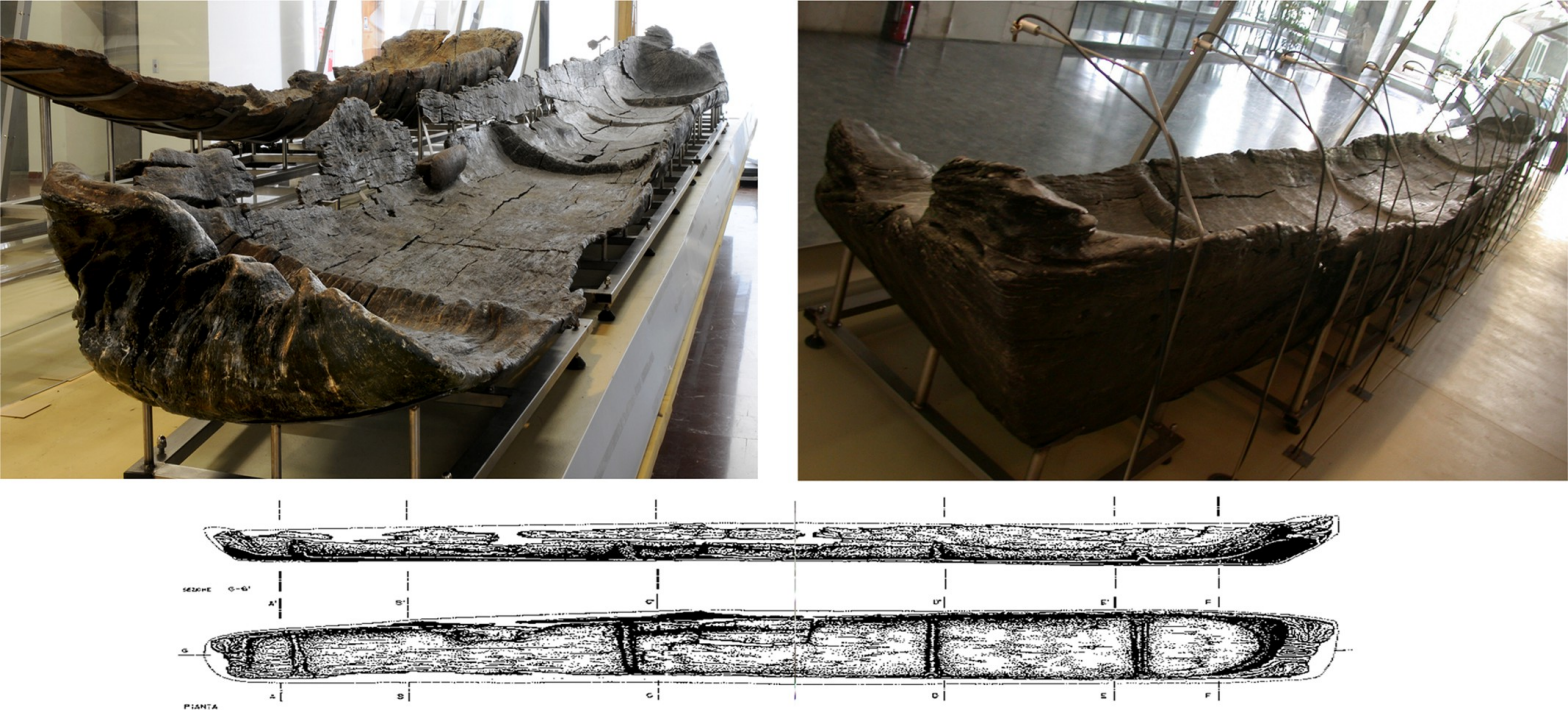
Canoe Marmotta 1. On display in the Museo delle Civiltà in Rome.

Canoe Marmotta 1 was found in Level II (Squares A62, A64, A108, A109, A110, A111, A112, A113, A153, A154, A155, A156 and A157). It is a huge dugout canoe made from an oak trunk (*Quercus* sp.) 10.43m long, 1.15m wide at the stern and 0.85m wide at the bow. It is 65 to 44cm high, depending on the part of the canoe. On the base of the canoe, four transversal reinforcements were made out of the same trunk with a trapezoidal shape. They would have increased the durability of the hull and protected it, as well as improving its handling [40] (Fig 4).

**Fig 5:**
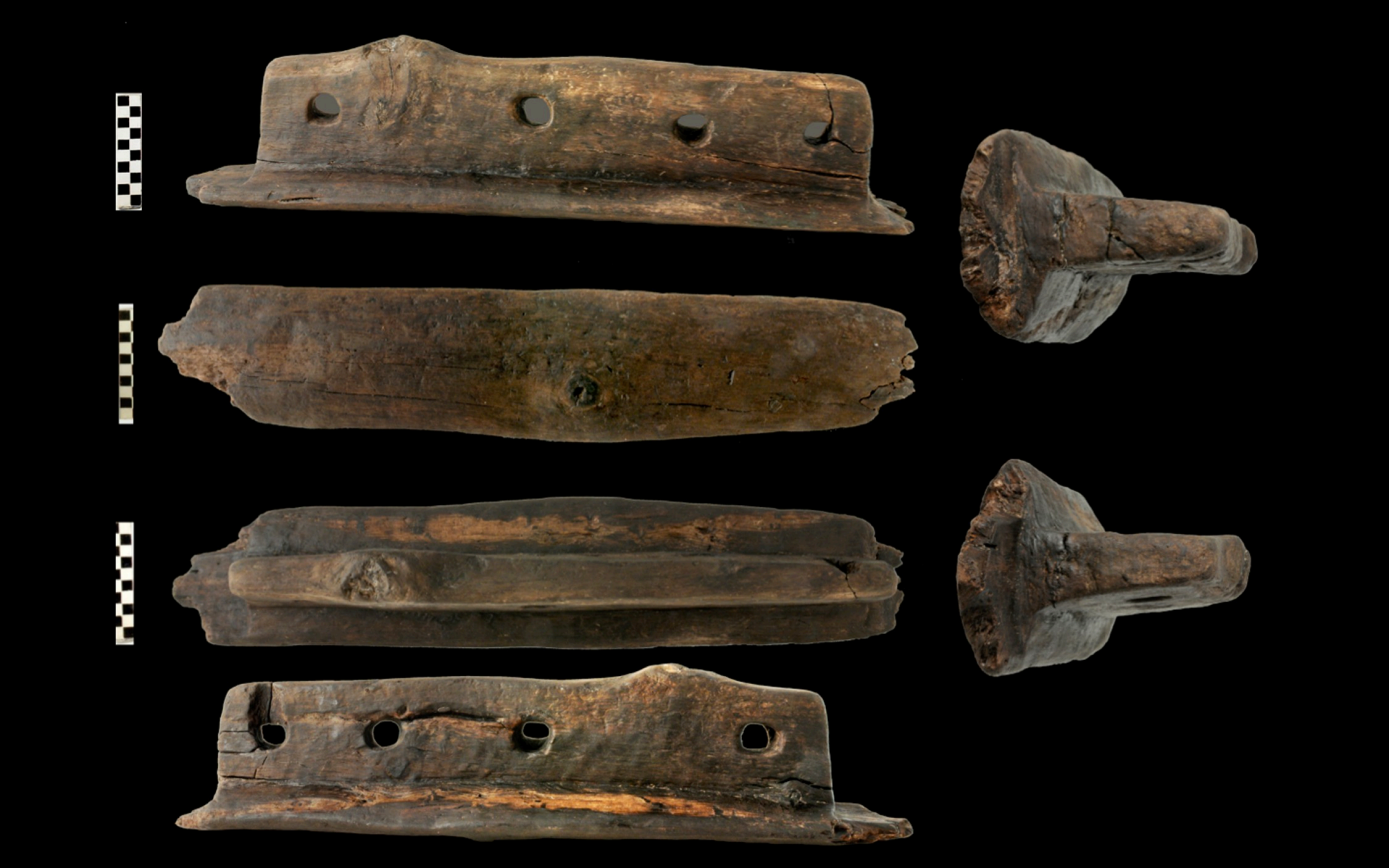
T-shaped element with four holes associated with canoe Marmotta 1. Object 144364, dated by ^14^C. On display in the Museo delle Civiltà in Rome.

In addition to its size, this canoe is of special interest because of three objects associated with its starboard side. They are T-shaped, with an ogival upper part, and with 2, 3 and 4 holes respectively (Fig 5). They were found inserted in the wall of the canoe at similar distances and heights. The visible holes were in the outer part of the canoe wall (Fig 5) Their size and shape are described elsewhere [40]. The characteristics and position of these objects suggest that they might have been used to fasten ropes tied to a possible sail or to join other nautical elements such as a stabiliser or even another boat to create a double hull in the form of a catamaran [44]. Those strategies would have provided greater safety and stability, and greater capacity for the transport of people, animals and goods [21].

**Fig 6:**
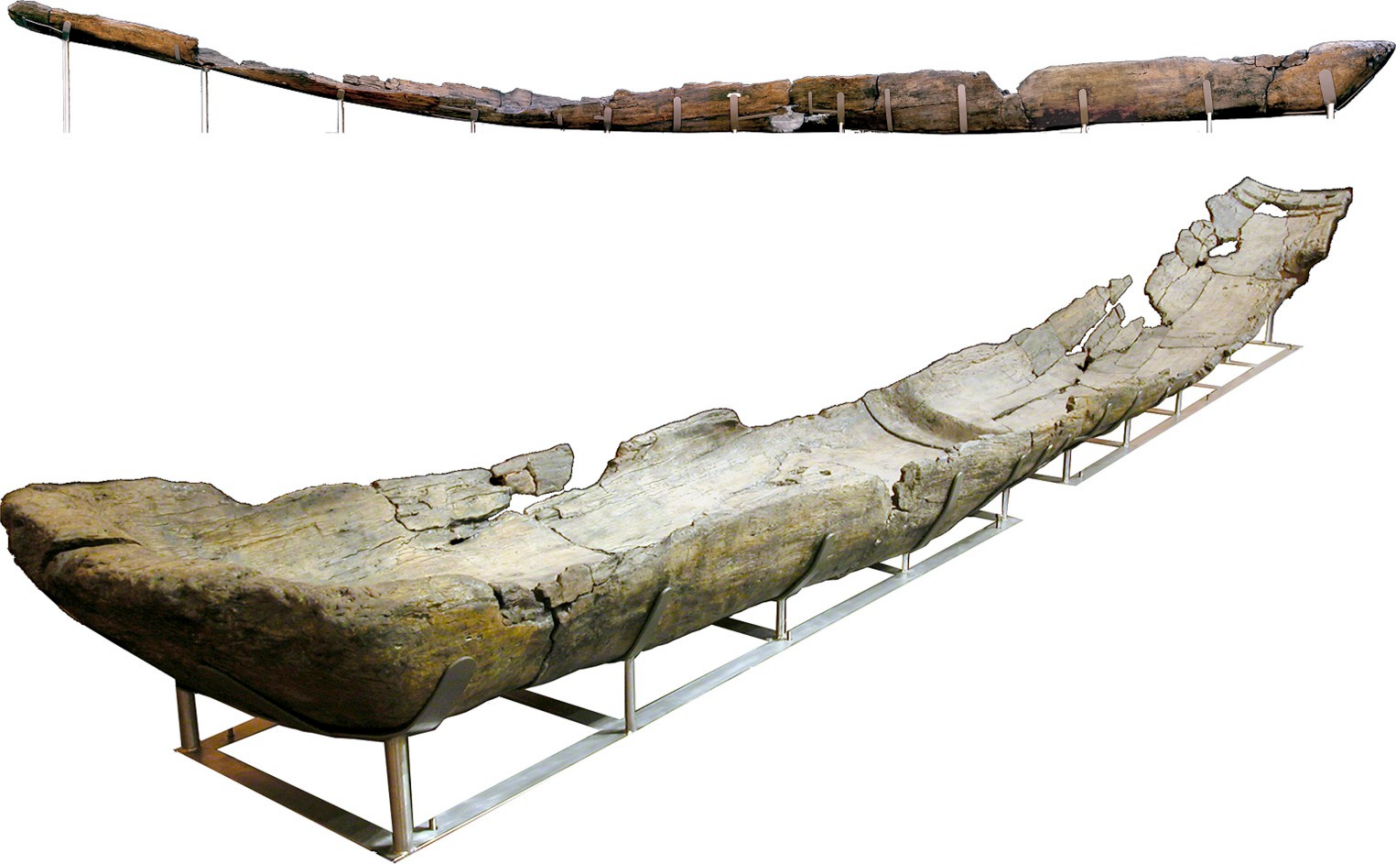
Canoe Marmotta 2. On display in the Museo delle Civiltà in Rome.

A large alder trunk (*Alnus* sp.) was chosen for the second canoe, Marmotta 2. Found in Level II (Squares D185, D186, D187, D186, D188, D237, D238 and D240), it was fastened to the ground with two sticks in the middle of the starboard and port sides (Fig 6). It is 5.4m long, 0.4m wide in the stern and 0.36m wide in the bow. Judging by its size and shape, it is thought to have been a fishing boat, or used to gather plant resources and transport people and small animals on the lake, or even on the sea [41].

A piece of wood with a single hole, about 2.8cm in diameter, was found with this canoe. It is mushroom-shaped, 13.4cm long and between 9.1 and 8.3cm wide. The characteristics of this object, and its similarity to modern bollards seen in our ports, suggest that its function might have been precisely that, to secure the canoe when the water level rose in the lake.

**Fig 7:**
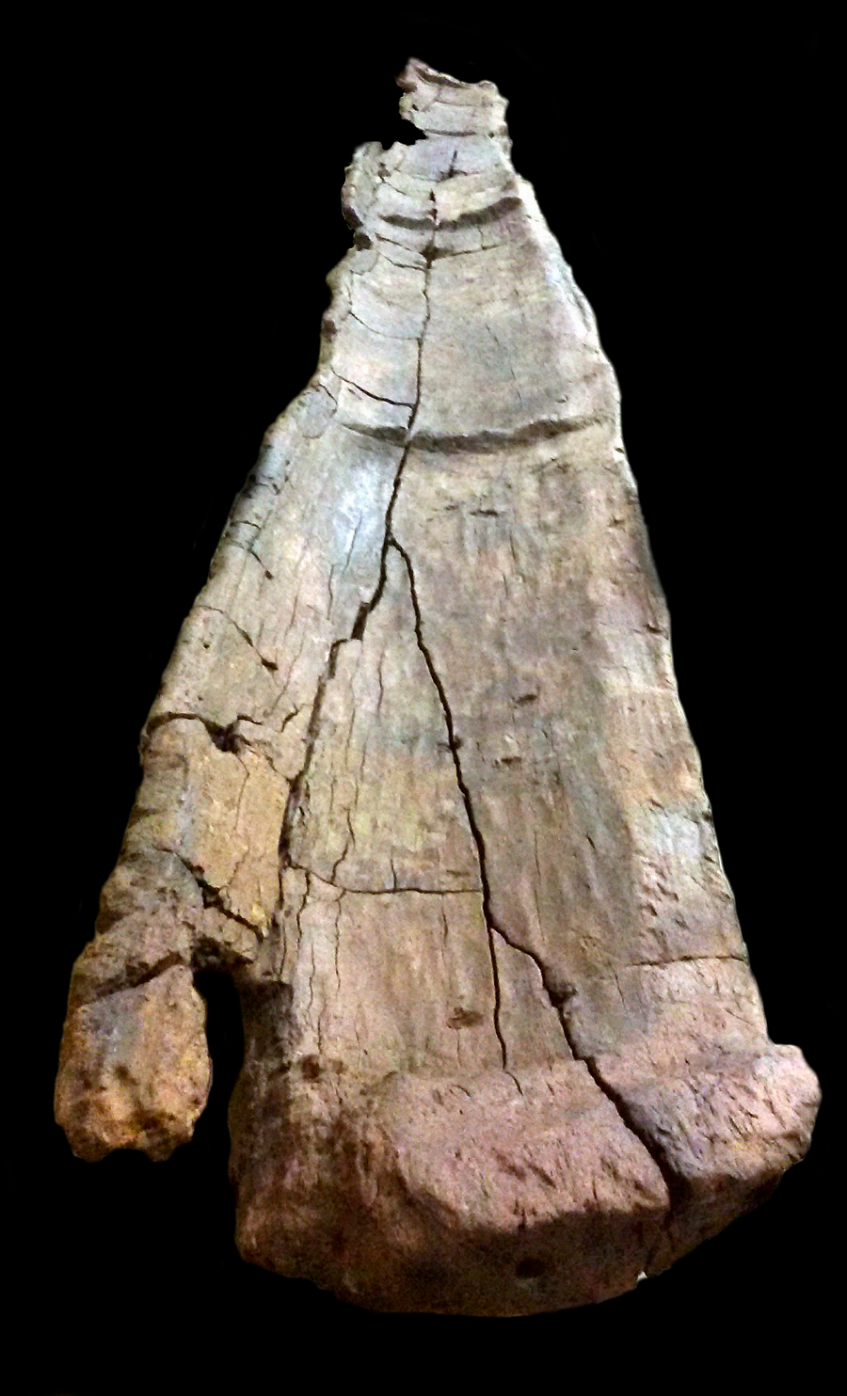
Canoe Marmotta 3. On display in the Museo delle Civiltà in Rome.

Canoe Marmotta 3 was found in Squares A136, A138, A140, A183, A185 and A187 (Levels I and II) (Fig 7). Made out of an alder trunk (*Alnus* sp.), it is 8.35m long, 58cm wide at the stern and 50cm wide at the bow. Like Canoe Marmotta 1, at the base, three transversal reinforcements made in the trunk itself are positioned at a similar distance and trapezoidal in shape. During the excavation a transversal fracture was observed 4m from the stern, which broke the canoe in two. This is probably a post-depositional fracture as a consequence of a natural action. This canoe was also on land as it was fixed to the ground with three sticks.

**Fig 8:**
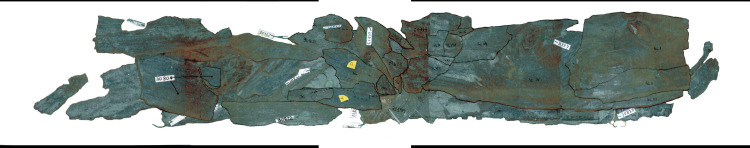
Canoe Marmotta 4. On display in the Museo delle Civiltà in Rome.

Canoe Marmotta 4 was discovered in Squares A263, A265, A266, A315, A316, A318, A367 and A368 (Fig 8). Owing to its size this dugout occupied the three levels: Chiocciolaio, Level I and Level II. Like the previous canoe, it was affected by a transversal fracture 4m from the stern. It was so badly deteriorated that much of the hull was missing. Made from a poplar trunk (*Populus* sp.), in its present fragmentary state it can only be said that its maximum width is 65cm. During the last stages of the excavation a large wooden board that might have formed part of the canoe was found on its port side.

**Fig 9:**
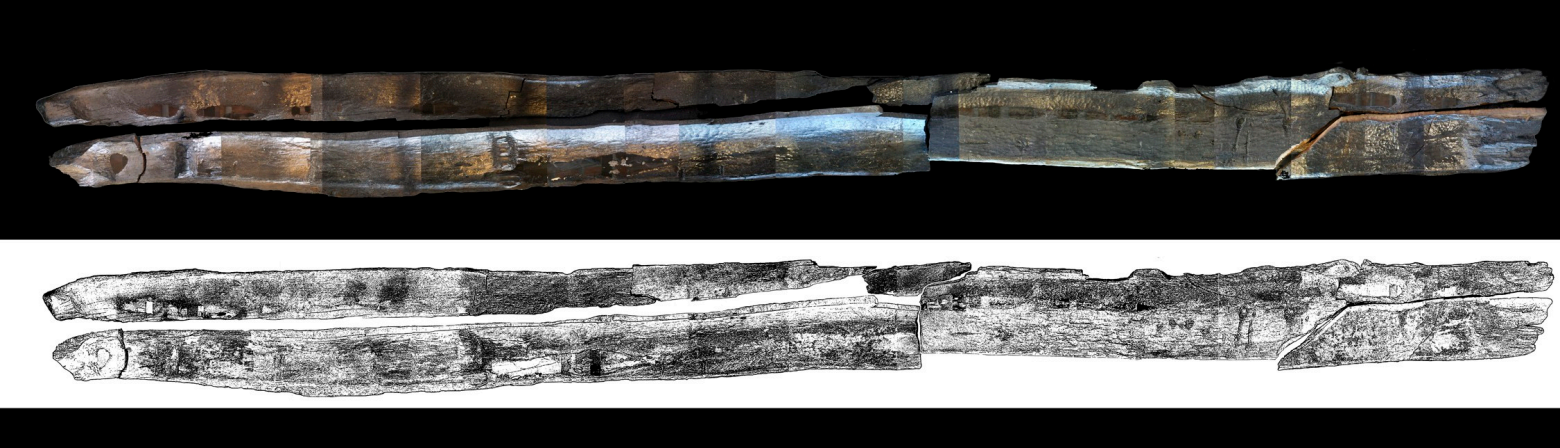
Canoe Marmotta 5. On display in the Museo delle Civiltà in Rome.

The last of the canoes, Marmotta 5, was excavated in Squares A401, A402, A403, A405, A354, A356, A367, A358, A359, A360, A361, D449 and D450 (Levels I and II) (Fig 9). Shaped from a beech trunk (*Fagus sylvatica*), in its present state it is 9.5m long and a maximum of 60cm in width in the area of the stern. These are not its real measurements because it is fragmented, and it obviously must have been larger. Two transversal reinforcements made in the trunk itself can be appreciated on the base of the canoe.

In addition, other objects documented at the site may have been associated with navigation. These are three wooden objects that might be oars or rudders.

- Object no. 11618 (Level I, Squares D287-D288) was found near canoe Marmotta 2. It is 105cm in length from the shaft to the blade.
- Object no. 4954 (Level II, Square A151) was retrieved near Marmotta 1. Although the proximal end of the shaft is broken, we can imagine its appearance. It is 44cm long in total; 16cm in the blade and 28cm in the shaft.
- The third of these objects is no. 12005 (Level I, Square D344). It is rectangular, between 17 and 19cm wide and 1.4 to 1.6cm thick. This blade must have been tied to the shaft with cord as six holes are aligned in the area of the connection, three on each side. This artefact was not found near any of the canoes as it came from a square between Marmotta 2 and Marmotta 5.

**Fig 10:**
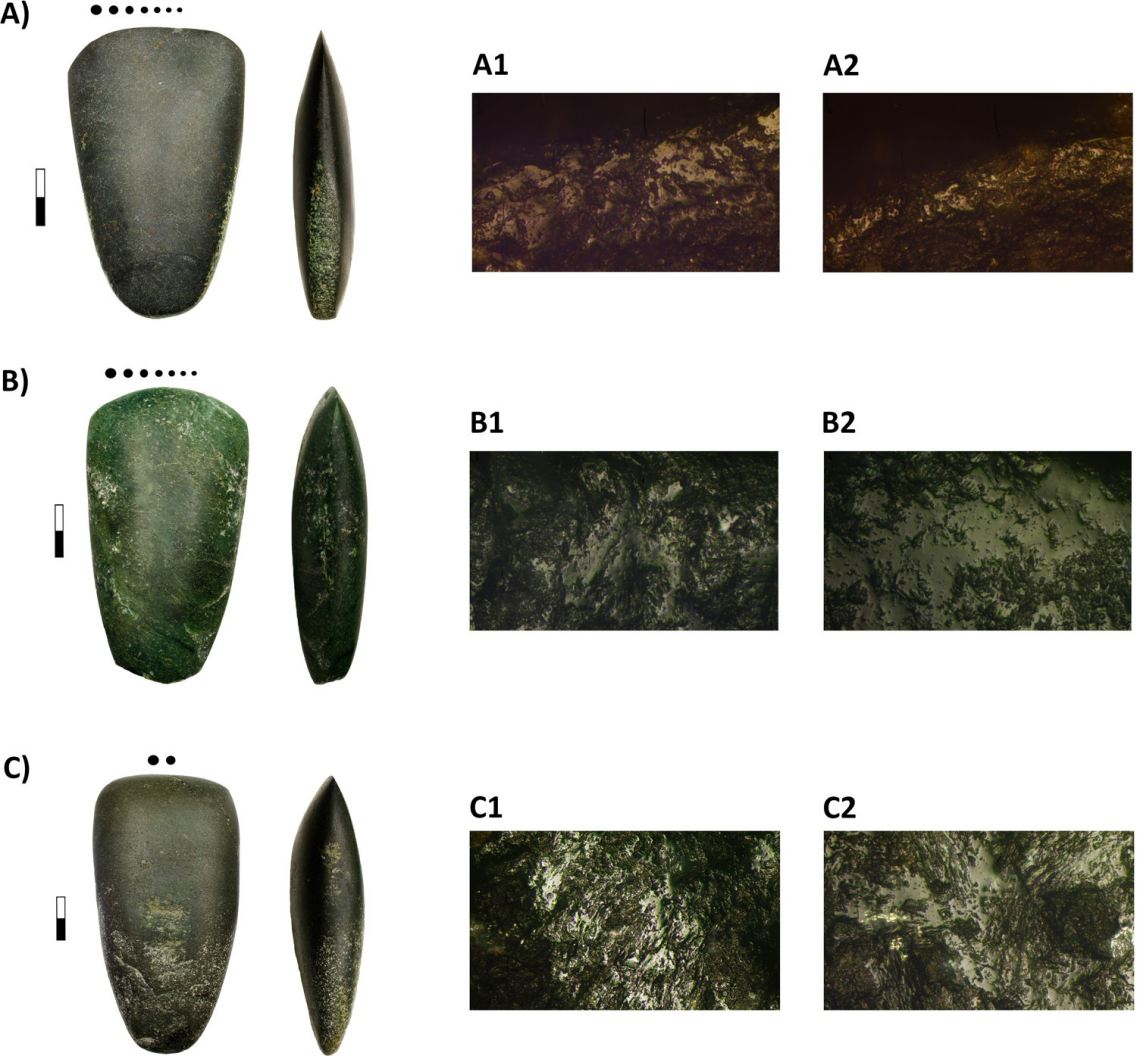
Stone adzes and axes displaying use-wear evidence of woodworking. A) tool 32454; A1-2) 200x; B) tool 40781; B1-2) 200x; C) tool 28881; C1-2) 200x (Picture: Alba Masclans).

The studies conducted on the canoes from La Marmotta show that they were carved with polished adzes and axes [40]. At La Marmotta, woodworking has been clearly attested by the use-wear study of the stone adzes and axes recovered in the excavations. Further use-wear analysis is currently being performed according to the methodological criteria established by Masclans (2020) [45] (Fig 10) and preliminary results have been recently published [46].

Given the important morphometric variability observed in the Marmotta stone adze record, it is to be expected that specialised tools were related to particular kinds of woodworking activities. Further experimental programs would be advisable to be able to discriminate between artefacts used for specific functions within woodworking, such as chopping down trees or other carpentry tasks, including canoe-making.

Like many other prehistoric boats in Europe, the work of carving them was simplified by burning their interior. This is clearly seen in Canoes Marmotta 1 and 4.

## 4. Methods

### 4.1 Botanical identification

The identification of the wood species was carried out by observing the three anatomical planes of the wood (transversal, radial longitudinal and tangential longitudinal). The samples were obtained by extracting three thin sections from each of the planes with a sharp instrument. Obtaining thin sections is an invasive technique and therefore the samples must be of a size appropriate for the identification of species, but not too large as to damage the object [47, 48]. The polyethylene glycol treatment (PEG) has not impeded the taxonomic study *a posteriori*, as the microanatomy of the wood can be observed, allowing taxonomic identification.

The thin sections were observed with an optic microscope equipped with objectives with 4, 10, 20 and 50x magnification (Leica DM 750M at the Instituto Patagónico de Ciencias Sociales y Humanas: IPCSH-CONICET) and compared with a specialized atlas [49, 50].

### 4.2 Radiocarbon dates

#### 4.2.1 Sample selection and processing

The whole procedure for radiocarbon dating of the wood samples was performed at the Centro Nacional de Aceleradores (CNA) in Seville, Spain. Samples were first chemically treated in the laboratory to remove contamination, and then were graphitized for AMS measurement.

The outsite of the trunks used to make the canoes was lightly quarried by the La Marmotta community itself. To minimise its impact on the dating, the outer part of the trunks, probably belonging to the most recent growth rings, was selected for the samples. In this way, we avoided ageing the making of the canoes with the age of the tree.

With the exception of canoe Marmotta 5, Sample CNA5349.1.1, the wood had previously been treated with polyethylene glycol (PEG), which must be considered a contaminant for radiocarbon dating. Thus, a proper pre-treatment method must be applied to remove PEG molecules from the wood structure. Such organic compounds are usually removed by organic solvent extraction in a Soxhlet apparatus, where the solvent in a glass container is heated, and the vapour is condensed in a cooling serpentine, dropping over the sample and dissolving the contaminant. Three consecutive organic solvents are used in increasing polarity order: hexane, acetone and ethanol, 15 minutes for each wash. Samples are dried in the stove after each wash at 80°C. After the organic solvent treatment, the usual Acid-Alkali-Acid procedure is applied [51].

A first acid wash (HCl, 0.5M) is applied at 80°C for 24 hours to eliminate remaining carbonates. The sample is then neutralized with Milli-Q grade water. A second alkali wash (NaOH, 0.1M) is applied to eliminate possible humic and fulvic acids, also at 80°C for 24 hours, and then the sample is neutralized. A final acid wash of only 15 minutes is applied to eliminate CO_2_ that may be absorbed from the atmosphere during the alkali wash. A final neutralization is performed, and the sample is dried in an oven overnight. At this point the wood sample is ready to be graphitized.

However, experience has shown that aged PEG may be very difficult to remove completely from wood even by those chemically aggressive procedures [52]. The effect of PEG on the date of a sample is not straightforward to assess and will depend mainly on three factors: the amount of residual PEG after the cleaning procedure, the ^14^C concentration of PEG (the “age” of the PEG), and the real age of the sample itself. This issue will be discussed in the following section.

Clean and dry samples are graphitized using the so called Automatic Graphitisation Equipment (AGE) [53]. About 3 mg of wood sample are combusted in an elemental analyser, where the combustion gases are separated, and in the case of CO_2_ injected into the AGE system. An amount of CO_2_ gas corresponding to 1 mg of carbon is kept in a reactor and is mixed with hydrogen and heated to 580°C in presence of iron as a catalyst. As a result, CO_2_ is reduced to graphite, and this is deposited over the catalyst. The mixture of iron and graphite is pressed in an aluminium piece to obtain the AMS target.

AMS targets were measured using a Micadas system [54]. In the AMS measurement the concentrations of the three carbon isotopes, ^12^C and ^13^C, which are stable, and ^14^C, which is radioactive, are determined, since all of them are needed to obtain the age of the sample. The final analysis of the data is performed using the BATS tool [55] to obtain the final radiocarbon ages and δ^13^C values, which are also measured in the AMS system. The δ^13^C parameter indicates the relative concentration of the stable isotopes and is used to correct the fact that different coetaneous materials contain slightly different radiocarbon concentrations. δ^13^C is included in the calculation of the radiocarbon age, which is performed as defined by Stuiver and Polach [56], and is the experimental result of the dating process.

Samples in this study were measured in different batches. Each batch contains unknown samples, standard samples for normalization, blank samples used for background correction, and one reference sample, IAEA-C8 [57] used to control the quality of the measurement. Consensus value of this material is pMC = 15.03±0.17 (one sigma). The pMC is a measure of the quantity of radiocarbon in a sample, expressed as the percentage of radiocarbon in the sample related to the defined standard [56]. The pMC results obtained for the C8 quality control samples in the different batches were 14.80±0.16, 15.03±0.08, 14.97±0.09 (one sigma), in agreement with the consensus value.

#### 4.2.2 Statistical analysis of the dates

Six new radiocarbon dates have been obtained: one for each of the five canoes and the other for one of the T-shaped objects associated with Canoe 1, the one with four holes (no. 144364) (Table 1).

**Table 1:** Radiocarbon dates of the five canoes and the nautical “T”-shaped object (144364).

Context	Lab.Code	^14^C Date	St.Dv.	Sample	Cal. BC (2σ)
Canoe “Marmotta 1”	CNA-5292.1.1	6220	39	*Quercus*	5305–5045
Canoe “Marmotta 2”	CNA-5898.1.1	6315	32	*Alnus*	5360–5215
Canoe “Marmotta 3”	CNA-5899.1.1	6613	32	*Alnus*	5620–5480
Canoe “Marmotta 4”	CNA-5348.2.1	6616	29	*Populus sp*.	5620–5480
Canoe “Marmotta 5”	CNA-5349.1.1	6461	42	*Fagus sylvatica*	5480–5330
144364 “T” shaped object	CNA-5900.1.2	6379	31	*Alnus*	5470–5225

The wood of five of the samples had been treated with PEG. Infra-red spectrometry was used to determine whether the cleaning process to remove the PEG had been effective. The results showed that the procedure had greatly reduced the presence of PEG in the samples, but not always completely. If PEG is synthesised with fossil products and contemporary organic products, it can alter the age of the sample (older or younger, respectively). However, in the present study, the dates obtained for all the samples are consistent with age of the settlement established by dating samples of seeds, charcoal and Canoe 5, which was not treated with PEG. This suggests that the effect of PEG in the present samples is, at most, very small.

All of them were analysed through different kinds of statistical tools in order to determine the chronology of the boats, as well as their degree of contemporaneity. These analyses were carried out using the OxCal v.4.4 software [58] and the IntCal20 calibration curve [59].

First of all, the temporal distribution of the canoes was determined from a Single Contiguous Phase Bayesian Model [58, 60, 61]. It is a simple uniform distribution model based on the hypothesis that all events (radiocarbon dates) have the same probability of occurring at any time between the beginning and the end of the phase. The model takes the oldest and most recent dates as temporal boundaries. If it presents high consistency with the data, it could be inferred that the dated events are distributed continuously throughout the entire phase.

The contemporaneity between the different boats was tested through the Chi-Square Test (OxCal Combine function). This test assesses the statistical consistency of the radiocarbon dates probability intervals overlap. Specifically, it estimates the mean of the set of dates and compares it with each of them individually. The test also estimates a total overall error by combining the standard deviations of the analysed radiocarbon determinations. If the result is statistically consistent, the contemporaneity of the pooled dates can be interpreted [62, 63].

Finally, the new set of dates was compared with a total of 58 available ^14^C dates for European boats between 7200 and 1700 cal. BC to place their temporality on a European scale by a Single Continuous Phase Bayesian Model (S1 File). Dates with standard deviations greater than ±75 were discarded from the analysis to restrict and specify as much as possible the probability intervals.

## 5. Results

### 5.1 Woody raw material

The taxonomic analysis showed that four different trees were used to make the five dugout canoes and the four-hole artefact found next to Canoe 1.

Canoes 2 and 3 and the artefact (no. 144364) were made of wood from *Alnus* sp. Deciduous *Quercus* sp. was used for Canoe 1, *Populus* sp. for Canoe 4 and *Fagus sylvatica* for Canoe 5. The heterogeneous use of wood determined by the taxonomical study does not support the selection of wood types with special properties or characteristics for the manufacture of the canoes from the La Marmotta archaeological site (Table 1). This taxonomic diversity is important because it shows the knowledge that the boat-builders had knowledge of the qualities of the wood and they knew which trees could be used to make the dugouts. The Quercus timber is characterized by considerable density and weight. It would provide tough wood and resistant to decay and available in different lengths and diameters. The advantage of Quercus is the presence of tyloses within the vessels, which lessen the permeability of its wood. The use of deciduous oak wood is generally well-documented at the site [28, 36]. The lightness of Alnus wood together with its resistance to splitting and cracking could have been an advantage for its use.

In contrast, at other sites where more than one canoe has been found, the same species was usually used for all of them. Thus, for instance, whereas alder was used at Ogårde, lime was used at Tybrind Vig and oak at Paris-Bercy [20, 64].

The archaeobotanical study of the canoes of the Neolithic site of La Marmotta, allows us to understand, on the one hand, the strategies of management and use of woody resources as raw material by the inhabitants of this village. On the other hand, this study allows to understand the level of specialization of the first Neolithic communities that spread in the Mediterranean.

### 5.2 Chronological analysis

**Fig 11:**
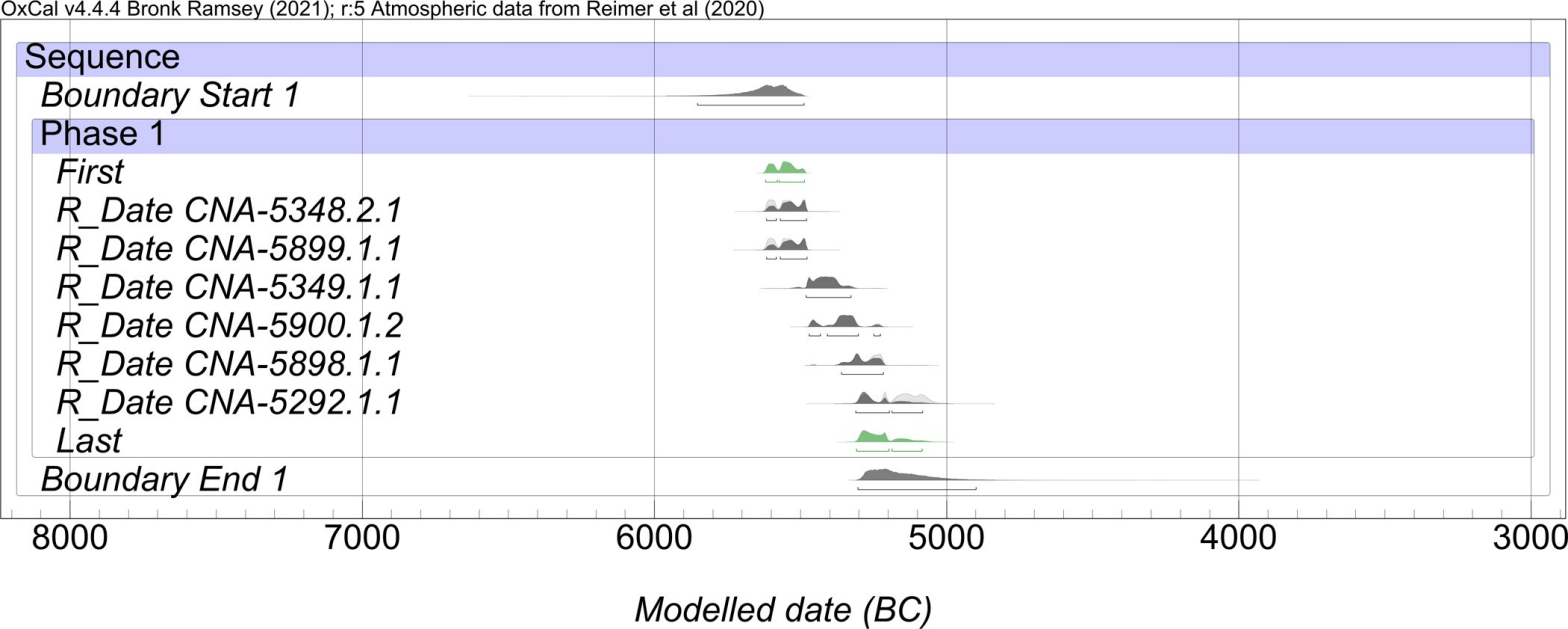
Chronology of the radiocarbon dates associated with the canoes from La Marmotta proposed by a Single Contiguous Phase Bayesian Model.

The Bayesian Model (Fig 11) determined that all the canoes and the other nautical object were distributed continuously in a single chronological phase (*Amodel 92*.*2* and *Aoverall*. *92*.*1)* between 5875–5490 and 5305–4860 cal. BC (95.4% probability) with a span of 205–490 years (95.4%).

The proposed boundaries for the model have a long tail because of the small set of modelled dates. The phase Model assumes that a random sample of the events was dated within the phase, so the long boundaries indicate that other unsampled events could be significantly earlier or later [58]. Thus, based on the available data, the first and last parameters (highlighted in green in Fig 11), are more reliable chronological intervals. According to them, the five canoes were continuously distributed in a more restricted chronological phase, between 5620–5490 and 5310–5085 cal. BC (95.4%, span of 200–485 years).

**Fig 12:**
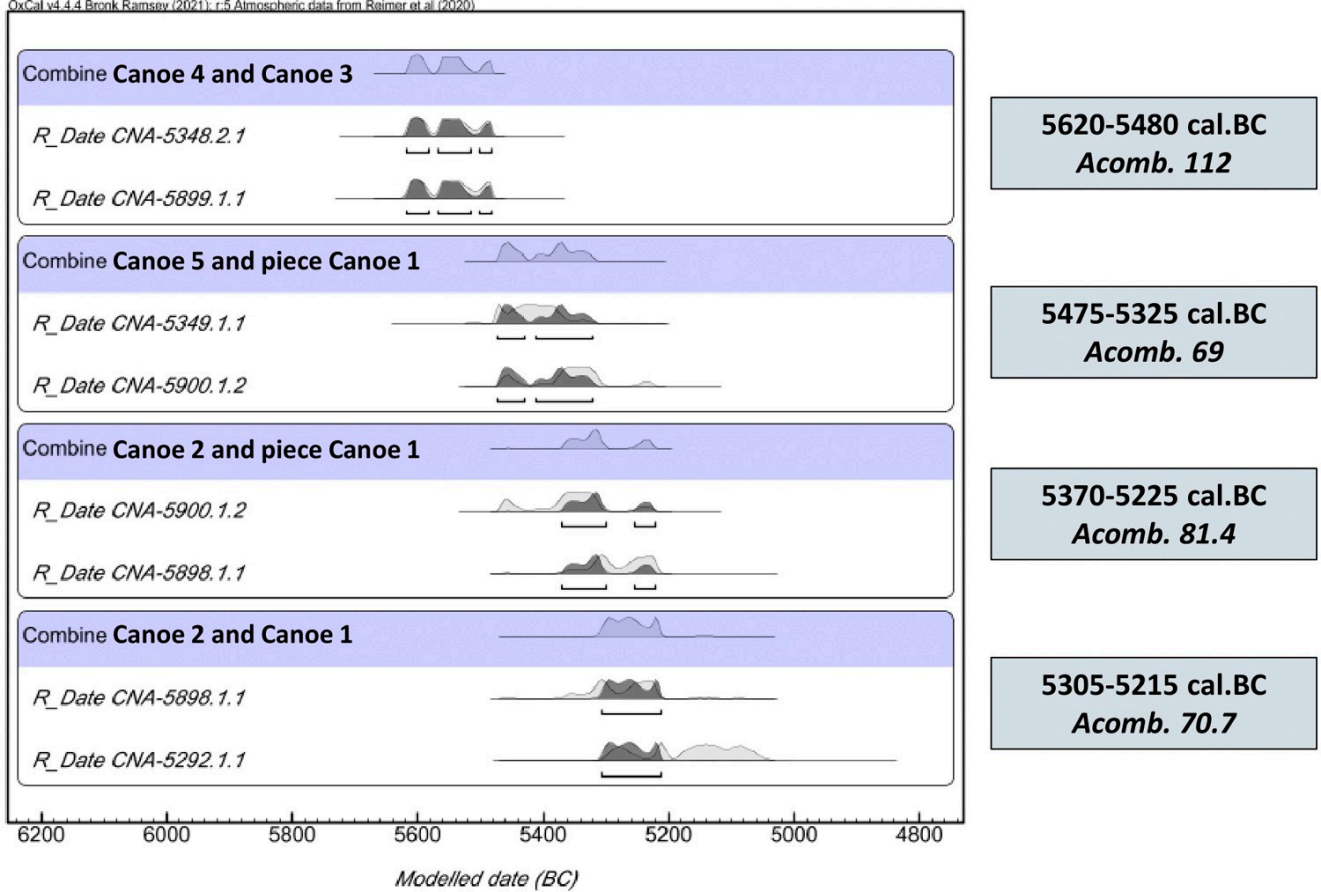
Contemporary canoes according to the Chi-Square test.

However, not all of them were contemporary during the chronological phase. Specifically, the OxCal’s Combine function (Fig 12) proposed that Canoes 4 and 3 were the oldest ones, being contemporary between 5620 and 5480 cal. BC (95.4%, Acomb. 112), while the object attached to Canoe 1 coincided in time with Canoe 5 between 5475 and 5325 cal. BC (95.4%, Acomb. 69) and with Canoe 2 between 5370 and 5225 cal. BC (95.4%, Acomb. 81.4). Finally, the latest boats were Canoe 2 and Canoe 1, which were synchronous between 5305 and 5215 cal. BC (95.4%, Acomb. 70.7).

The only canoe that is not contemporary with any of the other four boats is Canoe 5, whose calibrated intervals are dated in the middle of the chronology of the other boats (5480–5330 cal. BC). Canoe 5 is only contemporary with the element attached to Canoe 1 (5475–5320 cal. BC Acomb. 69). However, the radiocarbon date for Canoe 1 does not coincide with the date of this attached element. Instead, the Chi-Square test determined that the wood for Canoe 5 and that element were procured at approximately the same time, and that this element was subsequently attached to Canoe 1.

Despite being dated on long-lived samples, the probability intervals of the radiocarbon dates coincide with the chronology of La Marmotta that is based on the dating of both the wooden piles [30] and charred cereal seeds recovered from the archaeological layers (Levels 1 and 2) [39]. This demonstrates that the trees were felled directly with the purpose of building the boats and they did not come from previously felled or reused pieces. Moreover, the different contemporaneity relationships, as well as the short time of the continuous interval in which all of the canoes are chronologically distributed (around 200–480 years), demonstrates that they were built only over 4 or 5 generations.

**Fig 13:**
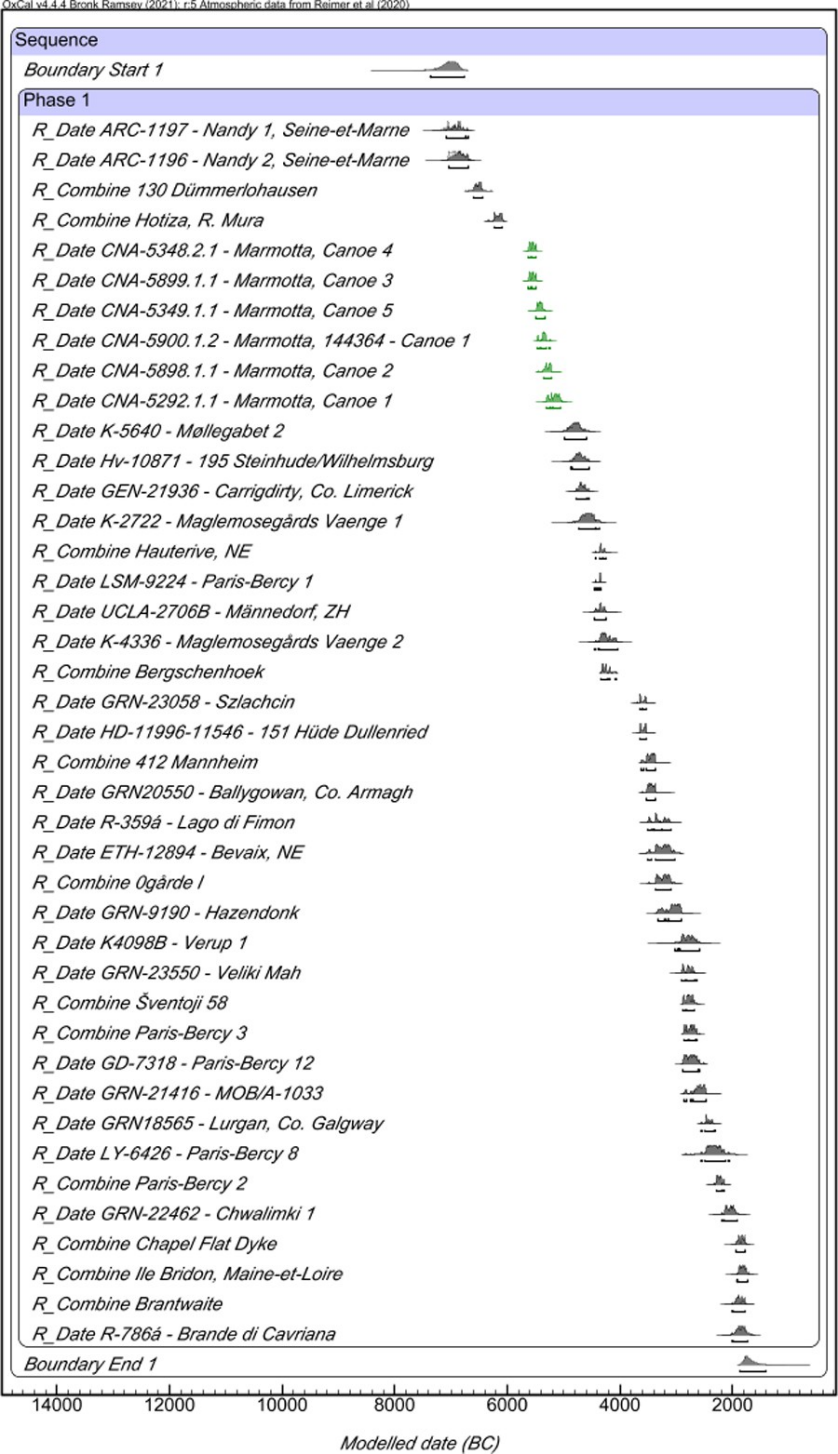
One Contiguous Phase Bayesian Model of European canoes.

The Bayesian Model (Fig 13) determined that the modelled European canoes were continuously distributed between 7360–6755 and 1865–1420 cal. BC (Amodel 95.3 and Aoverall 94.5) with a span of 5325–4930 years. Specifically, the boats from La Marmotta (highlighted in green) are among the 10 earliest dated so far in Europe. The Mesolithic Nandy 1 (7180–6705 cal. BC) and Nandy 2 (7060–6700 cal. BC) from Seine-et-Marne (France) are the oldest canoes, followed by the ones found in Dümmerlohausen (Germany, 6596–6445 cal. BC) and in Hotiza (Slovenia, 6240–6080 cal. BC). Therefore, the canoes at La Marmotta are the oldest known Neolithic boats.

As can be observed in S1 File, there was a preference for particular tree species that is unrelated to the chronology. This might be expected, since human communities have always made use of the species they found in their surroundings. This would be the case, for example, at Dümmerlohausen (*Alnus* sp.), Hauterive (*Tilia* sp.) and Paris-Bercy (*Quercus* sp.). The situation at La Marmotta is different however because no single species was employed for all the canoes and instead four trees were selected for the five boats (the same species, alder, was used for two of them, Canoes 2 and 3). This is indicative of the wide knowledge that the community at La Marmotta possessed about the properties of the trees they used to make their boats.

The dugout canoes from La Marmotta can be chronologically ascribed to a second, or a later, phase of the Italian Early Neolithic, which coincides with the expansion of Neolithic communities into the inland territories of South and Central Italy. The earliest dates for Impressed Ware in South Italy are *ca*. 6100–6000 cal. BC [66]. At that time, Neolithic communities were limited to southern Italy, mainly around the Gargano promontory and the Salento region, and to the Adriatic shores of Middle and Northern Dalmatia. Coastal expansion proceeded quickly; around 5900 cal. BC, sites such Arene Candide in Liguria, or Pont de Roque-Haute and Peiro Signado in the Gulf of Lyon were already occupied, confirming the importance of maritime navigation as rapid form of human dispersal. Terrestrial expansion appears to proceed more slowly, with a gap of at least a few centuries. Farmer groups gradually penetrated into Central Italy, in the Molise and Abruzzo regions (i.e., sites of Santo Stefano di Ortucchio, Monte Maulo) in about 5800 cal. BC. From 5600 cal. BC onwards, when the shores of Lake Bracciano were finally settled, Neolithic communities had already expanded further north, at San Marco di Gubbio (Umbria) and Portonovo Fosso Fontanaccia (Marche). The Po plain would be reached only later, between 5400 and 5300 cal. BC. The impact of riverways in this process of expansion is not clear, especially for peninsular Italy, a stretch of territory in which the main river basins are oriented on an east-west or west-east axis, although is probable that basins of large rivers such as the Tevere or the Po acted as vectors for inland diffusion. The diversity in size and shape of the Marmotta dugout canoes suggests that they were possibly used for both rivers and seafaring and, therefore, canoes like those at La Marmotta might have played a role not only for coastal expansion but also inland.

## 6. Conclusions

The dugout canoes at La Marmotta are undoubtedly exceptional examples of prehistoric boats and nautical systems. Their size, the other elements associated with them, and the variety of tree species make this site a compulsory point of reference in any discussion on the neolithisation process around the Mediterranean Sea and the origins of sailing.

These canoes at La Marmotta, and the occupation of many islands in the eastern and central Mediterranean during the Mesolithic and particularly the early Neolithic periods, are irrefutable proof of the ability of those societies to travel across the water. This is enormously significant, because all the canoes found at European Mesolithic and Neolithic sites are associated with lakes and therefore with sailing in those waters.

In the case of La Marmotta, the size of the lake (it is now 9.3km across, but must have been smaller in the Neolithic as the shore was 300m from its current position) barely justifies the large size of a canoe nearly 11m long. It is therefore possible that they were used to cover the 38km from Lake Bracciano to the Mediterranean Sea along the River Arrone. In this way, the canoes were used both in the lake and on the sea. Plentiful indirect evidence has been found of contact, and consequently voyages, between communities on different Mediterranean islands. In the case of La Marmotta, while the shape of some pottery recipients and the white ceramic figurine recall Greek and Balkan products, the obsidian used to make some lithic implements, especially some laminar tools, came from the islands of Lipari and Palmarola [38].

The seaworthiness of the canoes has been demonstrated by experimental archaeology. In 1998, in the framework of the project *The Sea Navigation in Early Neolithic Period*. *A Contribution of Experimental Archaeology to the Beginnings of Mediterranean Neolithization Monoxylon II*, Radomír Tichý‘s team built a reproduction of the canoe Marmotta 1 and sailed over 800km around the Mediterranean coast from Italy to the beaches of Portugal [65, 66]. Later, another canoe similar to Marmotta 1, ‘Monoxylon III’, was launched as part of the Navis project (https://projektnavis.com). The crew, with no experience of sailing, was formed by 8 to 10 rowers and a coxswain. They rowed in three hour shifts during most of the day and thus achieved an average speed of about 50km/day in favourable weather and sea conditions. If it can be supposed that Neolithic crewmen must have been more experienced sailors, they would surely have covered long distances in a short time, especially in the most suitable months. In any case, experimental archaeology is providing a clear picture of the extraordinary nautical skills possessed by members of the Neolithic community at La Marmotta.

Thus, there must have been people who knew how to choose the best trees, how to cut the trunk and hollow it by burning out its middle, and how to stabilise the dugout with transversal reinforcements on its base, or perhaps by the use of side poles or even parallel canoes in the form of a catamaran. To achieve this they made a series of amazingly modern artefacts, such as the T-shaped objects with two, three or four holes. These canoes and nautical technology are undoubtedly reminiscent of much more recent navigation systems. This shows that many of the major advances in sailing must have been made in the early Neolithic.

This technical complexity must be linked to social organisation in which some specialists were dedicated to particular tasks. The canoes can only be understood in a context of collective labour overseen by a craftsman who was in charge of the whole process: from cutting down the tree to launching the canoe in the lake or in the sea. These communities would therefore be well-structured in the organisation of labour, since collaboration would have been necessary to build the houses, make the canoes, procure raw materials from their sources several hundred kilometres away and put in practice certain agricultural tasks.

In this way, La Marmotta is causing a literal sea change in our view of those first Neolithic farming groups. It was always difficult to understand how they could have travelled around all Mediterranean Europe. The Marmotta canoes are not only outstanding evidence of how they achieved that but also an example of the complexity of those societies from the viewpoint of their social and technical organisation. These dugouts and the other elements associated with them represent the knowledge and experience accumulated over centuries and the practical skills of those groups to make them.

It is only in this way that we can understand how they crossed the Mediterranean Sea and occupied the coasts of Europe and Africa in the space of a few centuries. In the lands where they settled they introduced their new economic model based on domestic plant and animal species. That model has reached the present time and thus we are without doubt their most direct heirs.

## Supporting information

S1 File List of all 14C dates made on canoes. (XLSX)
